# Effects of Cr, W, and Mo on the High Temperature Oxidation of Ni-Based Superalloys

**DOI:** 10.3390/ma12182934

**Published:** 2019-09-11

**Authors:** Si-Jun Park, Seong-Moon Seo, Young-Soo Yoo, Hi-Won Jeong, HeeJin Jang

**Affiliations:** 1 Multi-Material Research Center, Gwangju-Jeonnam Division, Korea Automotive Technology Institute, 55 Jingok-sandan-jungangro, Gwangsan-gu, Gwangju 62207, Korea; sjpark@katech.re.kr; 2 High Temperature Materials Group, Korea Institute of Materials Science, 797 Changwondaero, Seongsan-gu, Changwon 51508, Korea; castme@kims.re.kr (S.-M.S.); yys@kims.re.kr (Y.-S.Y.); won680@kims.re.kr (H.-W.J.); 3 Department of Materials Science and Engineering, Chosun University, 309 Pilmundaero, Dong-gu, Gwangju 61452, Korea

**Keywords:** high temperature oxidation, alloying element, Ni-based superalloy, cyclic oxidation

## Abstract

The oxidation behavior of Ni–9.5Co–(8~12)Cr–(2.5~5.5)Mo–(4~8)W–3Al–5Ti–3Ta–0.1C–0.01B alloys was investigated at 850 °C and 1000 °C The mass change, the phase of oxides, and the cross-sectional structure of specimens were analyzed after cyclic oxidation tests. The oxide scale was composed mainly of Cr_2_O_3_ and NiCr_2_O_4_, but NiO, TiO_2_, and CrTaO_4_ were also found. Al_2_O_3_ was formed beneath the Cr oxide layer. The Cr oxide layer and internal Al oxide acted as barriers to oxidation at 850 °C, while Al oxide was predominantly protective at 1000 °C. Cr increased the mass gain after oxidation test at both temperatures. Mo increased the oxidation rate at 850 °C but decreased the oxidation rate at 1000 °C. W slightly increased the mass gain at 850 °C but did not produce a significant effect at 1000 °C. The effects of Cr, Mo, W, and the temperature were discussed as well as the volatilization of oxides, the valence number of elements, and diffusion retardation.

## 1. Introduction

Ni-based superalloys are used for heat exchangers, gas turbine blades, and chemical reactors because of their excellent oxidation resistance and mechanical strength at high temperatures. The oxidation behavior of superalloys is influenced by their chemical compositions. Cr and Al are the representatives of protecting alloying elements [1,2,3,4,5,6,7]. Cr forms a dense Cr_2_O_3_ layer on the surface of Ni-based superalloys and reduces the oxidation rate. It is very effective in protecting the alloy at temperatures up to about 871 °C (1600 °F). Above this temperature, Cr_2_O_3_ becomes unstable and volatile CrO_3_ forms. The role of Al is crucial at higher temperatures. When Al forms a dense and continuous layer of Al_2_O_3_, oxidation of the alloy is significantly reduced. The formation of the Al_2_O_3_ layer depends on the temperature and alloy composition [8,9,10,11,12].

Mo and W are known to reduce the oxidation resistance of Ni-based superalloys [13,14,15,16,17]. However, some reports indicate that these elements can stabilize the Cr_2_O_3_ layer [18,19]. The effects of Mo and W have been explained as promoting or hindering the formation of Cr_2_O_3_ or Al_2_O_3_, as well as the volatilization of their oxides [20,21,22,23,24,25,26,27].

Recent studies show that an alloying element can change the role of another element so that contradictory results are obtained depending on the alloy composition and temperature [8,28,29]. For example, increasing the Ta content in alloys with a low concentration of Al reduces the oxidation rate, but there is almost no effect in alloys with a high Al content [8]. Cr increases the mass gain of alloys with W during oxidation, but decreases the mass gain of the alloys without W [29]. Furthermore, Kim et al. [30] showed that a regression model from a Response Surface Model study predicted the rankings of several commercial alloys, although they failed to predict a reasonable oxidation rate for each alloy.

In this study, the oxidation behavior at 850 and 1000 °C of Ni-based superalloys containing Co, Cr, Mo, W, Al, Ti, and Ta as alloying elements is examined as a function of the Cr, Mo, and W contents. The effects of Cr, Mo, and W on the oxidation rate are determined, and the mechanism for these are discussed.

## 2. Experimental Procedures

Ni-based superalloys, with compositions listed in Table 1, were used as specimens. The alloys were cast into a button by vacuum arc melting. The buttons were turned over and remelted several times to make the composition homogeneous. The homogeneity was confirmed using energy dispersive spectroscopy (EDS, S-4800, Hitachi, Japan) on multiple points on the specimens. The specimens were then cut into disks of 10 mm in diameter and 4 mm in height and smoothed with a milling machine, followed by surface finishing with #2000 SiC abrasive paper. Each sample was contained in an alumina crucible of 20 mm × 20 mm × 15 mm in size and put into a box furnace (Jeiotech MF-GH32, Daejeon, South Korea). The alumina crucibles were preheated to 1000 °C for 100 h to ensure that the weight of the crucibles did not change during the oxidation test.

The oxidation test was performed for six cycles. Each cycle consisted of the following procedures: The specimens were put into the furnace when the temperature was 400 °C. The temperature was increased up to 850 or 1000 °C over 2 h and kept constant for 15 h. After thermostatic oxidation, the specimens were slowly cooled in the closed furnace with the power off for 2 h, and then in the furnace door open for about 2 h until the temperature reached 400 °C. The samples were retrieved at 400 °C and fully cooled in air.

The weight of the oxidized samples, including the fallen scales, was measured by a precision balance to an accuracy of five digits after the decimal point. The phase of the oxide scale was analyzed by using X-ray diffraction (XRD, X’pert Pro MPD, Malvern Panalytical, UK) on the oxide layer. The structure and the composition of the cross-section were examined by using scanning electron microscopy (SEM, S-4800, Hitachi, Japan) equipped with energy dispersive spectroscopy (EDS).

## 3. Results

**Figure 1:**
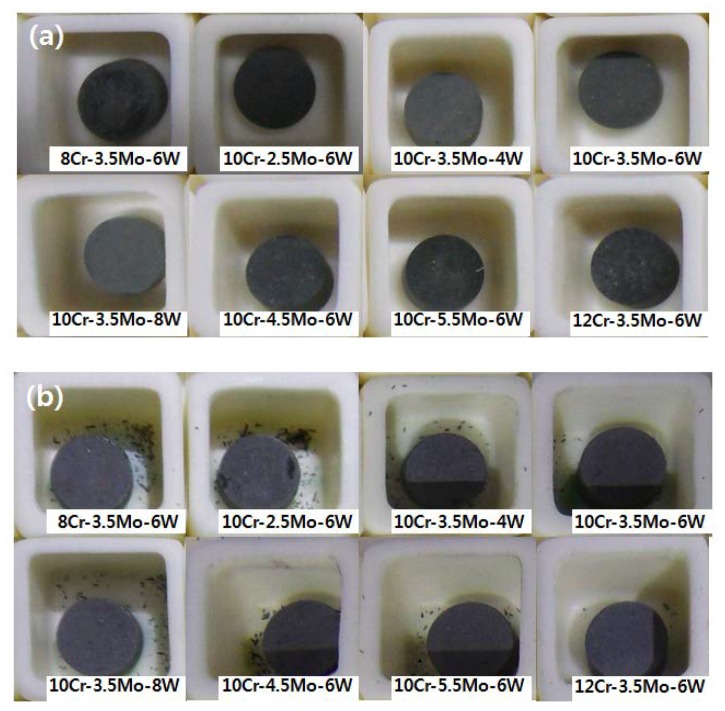
Specimens after six cycles of oxidation tests (**a**) at 850 °C and (**b**) at 1000 °C.

Figure 1 shows pictures of the specimens after oxidation test. The surfaces of the samples were dark gray in color. Oxidation was more severe at the higher temperature, as evidenced by spalled oxide particles around the specimens tested at 1000 °C.

**Figure 2:**
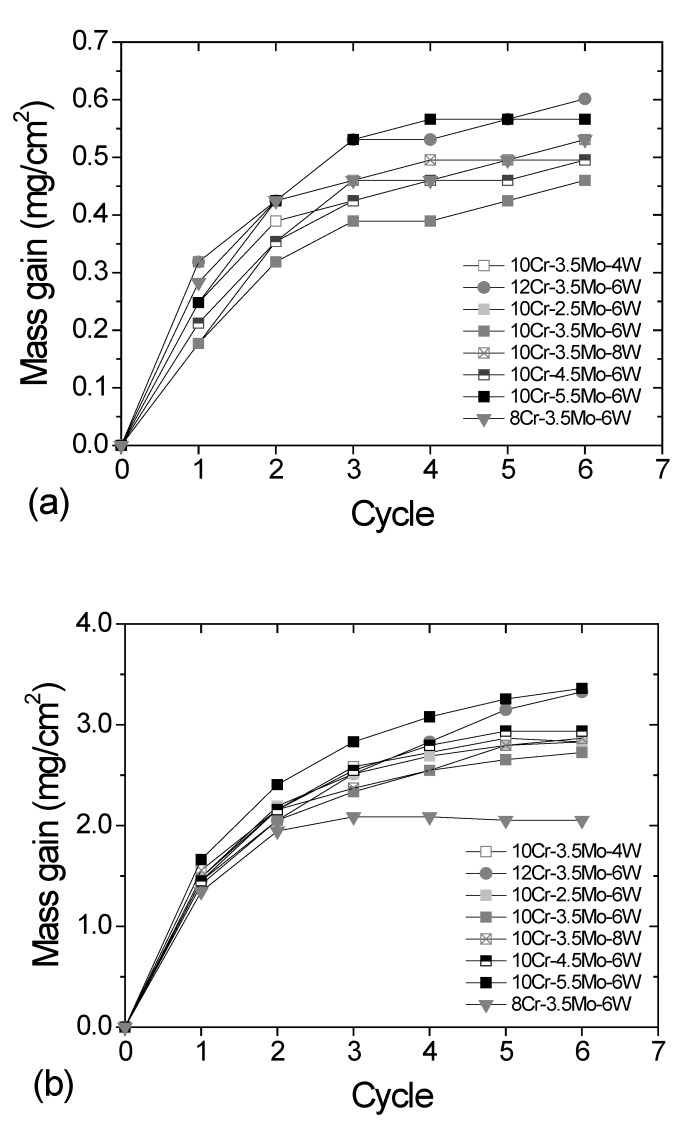
Mass gain during oxidation cycles (**a**) at 850 °C and (**b**) 1000 °C.

The weight of the alloys increased with oxidation, as shown in Figure 2. The mass gain after six cycles of oxidation at 850 °C was 0.46~0.62 mg/cm^2^. The oxidation rate was relatively high in the initial cycles and then gradually reduced with successive cycles. The weight of a specimen increased quickly when the oxide layer formed on the bare surface. The oxidation rate slows down after the initial stage, because the rate of oxide growth is controlled by the diffusion of oxygen and alloying elements in the oxide scale, as implied by the parabolic trend of the curves. The final mass gain at 1000 °C was 2.0~3.4 mg/cm^2^, much higher than that measured at 850 °C. The mass gain curves indicate continued growth of the oxide scale, except in the case for the 8Cr–3.5Mo–6W alloy at 1000 °C. The mass gain of 8Cr–3.5Mo–6W increased up to the third cycle but thereafter it became nearly constant. This is thought to be related to the volatilization of Cr hexavalent oxide. Severe spallation of scale on 8Cr–3.5Mo–6W, as seen in Figure 1, may also be a reason that no further increase of mass gain. Because we did not use any cover on the alumina crucible, part of the spalled oxide could have been lost during the tests.

**Figure 3:**
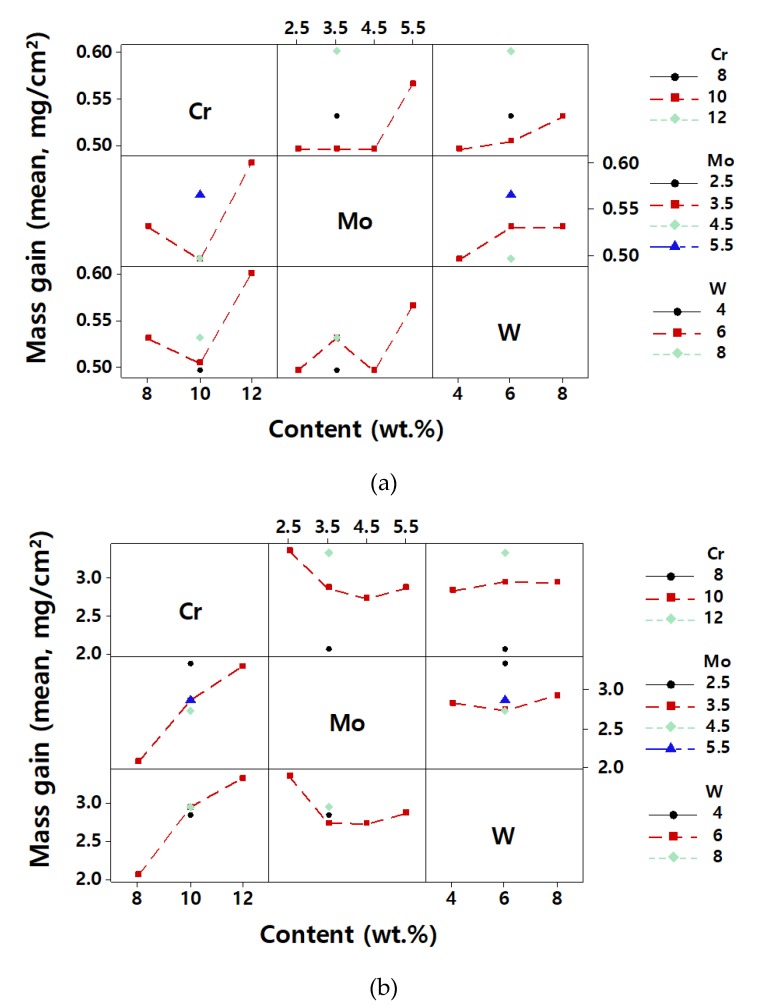
Final mass gain as a function of Cr, Mo, and W contents (**a**) at 850 °C and (**b**) at 1000 °C.

The effects of the alloy composition on the mass gain by oxidation are summarized in plots in Figure 3. Figure 3 shows the averaged mass gain of the samples as a function of the content of each element. The mass gain slightly decreased when the Cr content increased from 8 wt.% to 10 wt.% but it increased when Cr the content was increased to 12 wt.% (Figure 3a). Mo shows similar behavior in that the mass gain increased with an increase of Mo to over 4.5 wt.%. W increased the mass gain very slightly. The plots for 1000 °C (Figure 3b) suggest more clearly that Cr, Mo, and W increase the mass gain and the dependence is consistent over the composition range of this study. This result suggests that the three alloying elements do not suppress the oxidation of Ni-based superalloys effectively.

**Figure 4:**
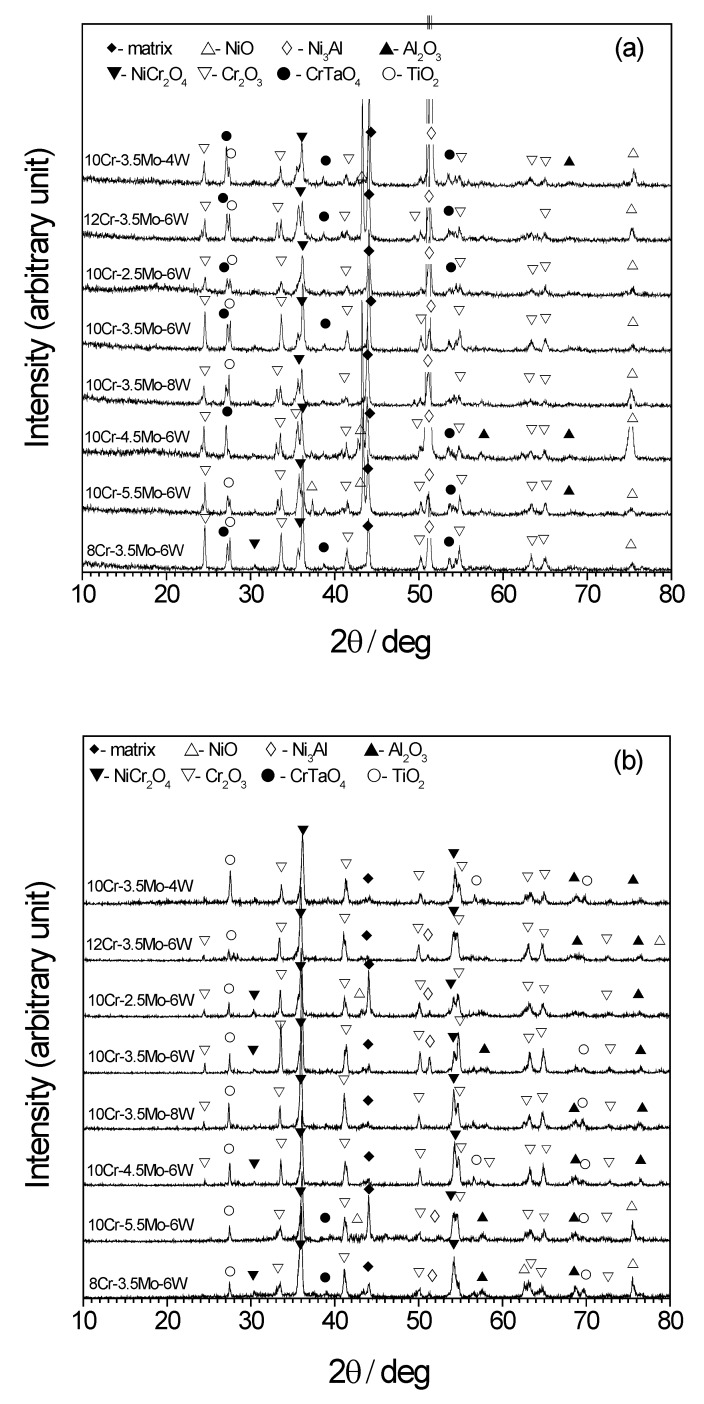
XRD pattern for the superalloys after oxidation tests (**a**) at 850 °C and (**b**) 1000 °C.

The oxide scale formed at 850 °C appeared to mainly consist of Cr_2_O_3_ and NiCr_2_O_4_ from the XRD pattern (Figure 4a). NiO, TiO_2_, and CrTaO_4_ were also detected. The peaks for Al_2_O_3_ were barely measured. At 1000 °C (Figure 4b), peaks for metallic phases (matrix and Ni_3_Al) were not measured, implying that the oxide scale is thick. The peaks for NiCr_2_O_4_ were the strongest among the studied oxide peaks at 1000 °C. The number and intensity of the Cr_2_O_3_, CrTaO_4_, and NiO peaks decreased significantly, while the peak for TiO_2_ seemed to be similar to the XRD patterns for 850 °C. Al_2_O_3_ was detected more frequently in the data for 1000 °C than in the data for 850 °C. The difference in the XRD patterns confirms that Cr_2_O_3_ is predominant in the scale formed at 850 °C and becomes unstable at 1000 °C.

**Figure 5:**
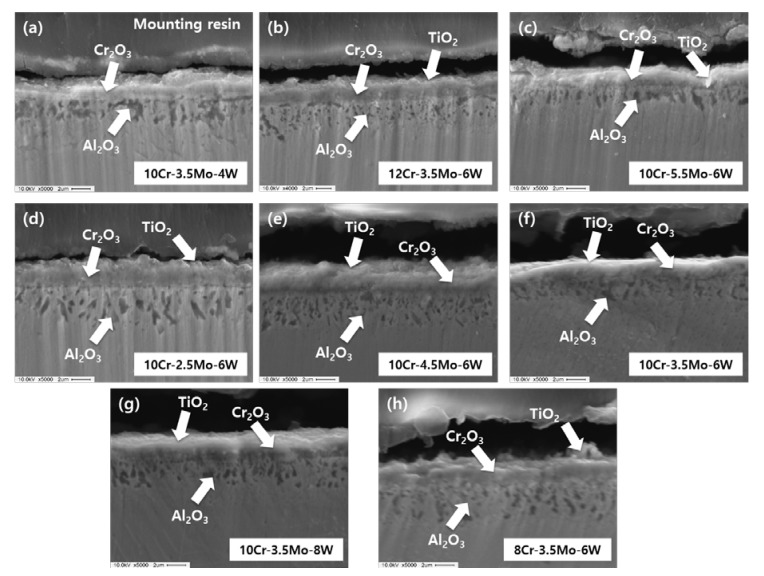
Cross-sectional image of (**a**) 10Cr–3.5Mo–4W, (**b**) 12Cr–3.5Mo–6W, (**c**) 10Cr–5.5Mo–6W, (**d**) 10Cr–2.5Mo–6W, (**e**) 10Cr–4.5Mo–6W, (**f**) 10Cr–3.5Mo–6W, (**g**) 10Cr–3.5Mo–8W, (**h**) 8Cr–3.5Mo–6W alloys oxidized at 850 °C.

The thickness of the oxide scale for the samples oxidized at 850 °C was several micrometers, as shown in Figure 5. The scale layer was mainly composed of Cr_2_O_3_ and discontinuous particles of Al_2_O_3_ were present just beneath the Cr_2_O_3_ layer. TiO_2_ was often found at the outermost part of the scale.

From the images of the (8–12)Cr–3.5Mo–6W alloys shown in Figure 5b, f and h, it is noted that the size of Al_2_O_3_ particles was larger in the alloys with lower Cr content. The high Cr content and the relatively low temperature as 850 °C are thought to be favorable for preferential formation of a stable Cr_2_O_3_ layer and hence Al_2_O_3_ might grow slowly due to the low diffusion of oxygen. The mass gain (Figure 3a) decreased with the increase in Cr content up to 10 wt.%, beyond which it then increased. These results imply that the oxidation resistance depends largely on the protectiveness of the Cr oxide layer at 850 °C but Al oxide plays an important part as well.

Figure 5c–f show the images for 10Cr–(2.5–5.5)Mo–6W alloys. The distribution depth of Al oxides tended to be smaller for the alloys with a higher Mo content, though it was not a linear function. It is anticipated that inward diffusion of oxygen is impeded by Mo, therefore the penetration depth of oxygen is limited in high Mo alloys. Mo is a high valence ion of Mo^4+^ or Mo^6+^, so it consumes more oxygen than Cr or Al. Mo is assumed to be incorporated or to form a small amounts of Mo oxide, as it was not detected by XRD (Figure 4). Since more Mo in the Cr_2_O_3_ layer bonds with more oxygen, the amount of oxygen which can diffuse into the region beneath the Cr_2_O_3_ layer is reduced in the alloys with higher Mo content. Consequently, decreased formation of Al_2_O_3_ results in increased mass gain during oxidation.

The effect of W can be seen in Figure 5a, f and g. The thickness and morphology of oxides do not seem to be so different for the three alloys, except that Al_2_O_3_ is a little continuous or large in the alloy with low W content (10Cr–3.5Mo–4W in Figure 5a). This result can be understood similarly to the case of Mo, in that W is also a high valance element that becomes W^4+^ or W^6+^ ion. Therefore, W can inhibit the diffusion of oxygen through the scale effectively. In addition, W is large in size and can retard the diffusion of other atoms nearby and the formation of internal Al_2_O_3_ is reduced, causing a relatively low oxidation rate of the alloy.

**Figure 6:**
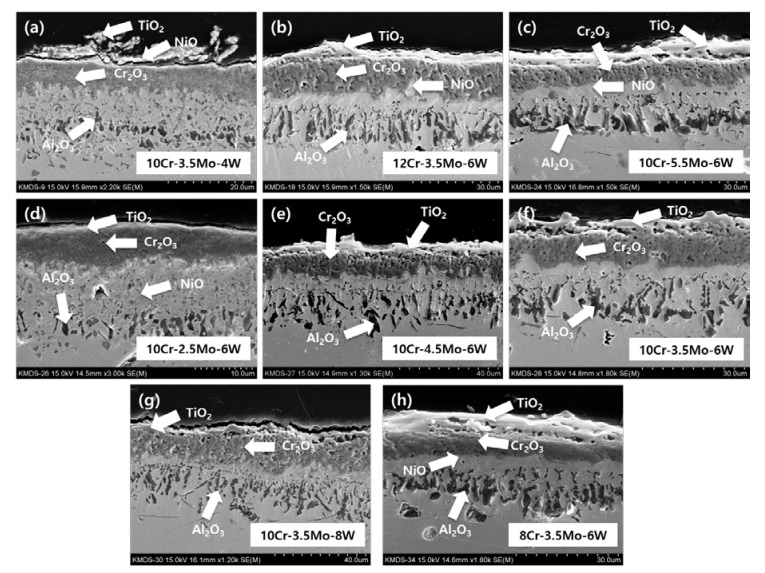
Cross-sectional image of (**a**) 10Cr–3.5Mo–4W, (**b**) 12Cr–3.5Mo–6W, (**c**) 10Cr–5.5Mo–6W, (**d**) 10Cr–2.5Mo–6W, (**e**) 10Cr–4.5Mo–6W, (**f**) 10Cr–3.5Mo–6W, (**g**) 10Cr–3.5Mo–8W, (**h**) 8Cr–3.5Mo–6W alloys oxidized at 1000 °C.

Figure 6 shows that the thickness of the oxide scale formed at 1000 °C was about 10 times thicker than that formed at 850 °C. The Cr_2_O_3_ layer formed at 1000 °C was porous and some cracks or voids are present between the Cr_2_O_3_ layer and TiO_2_ or NiO. Internal Al_2_O_3_ lumps had a more columnar shape than those formed at 850 °C. It can be interpreted that the stability of Cr_2_O_3_ is poor at 1000 °C and some Cr oxide were lost by volatilization of CrO_3_, leaving pores in the oxide layer. The porosity would increase the diffusion rate of oxygen; thus, the Al oxide could be formed at a deeper location. Diffusion of Al would also be greatly promoted at 1000 °C, so the size of Al_2_O_3_ would grow faster than at 850 °C.

We discuss the effects of the Cr content on the oxide structure, as shown in Figure 6b, f and h. The shape of Al_2_O_3_ became slightly thinner with a higher Cr content. A high Cr content suppresses the penetration of oxygen by forming Cr oxides and hinders the formation of Al_2_O_3_. However, Cr_2_O_3_ is not stable at 1000 °C and it transforms into volatile CrO_3_, leaving pores in the scale, or into NiCr_2_O_4_. NiCr_2_O_4_ is formed by the Cr_2_O_3_ + Ni + ½ O_2_ → NiCr_2_O_4_ or Cr_2_O_3_ + NiO → NiCr_2_O_4_ reaction [31,32,33,34,35,36]. This reaction involves the reduction of Cr_2_O_3_ and NiO, as confirmed by the XRD patterns in Figure 4. The protectiveness of the oxidation scale deteriorates due to the loss of Cr_2_O_3_ layer and insufficient formation of Al_2_O_3_, and the mass gain increased with the increase of Cr content as shown in Figure 3b.

For the 10Cr–(2.5–5.5)Mo–6W alloys (Figure 6c–f), the porosity of the Cr_2_O_3_ layer was notably higher in the 10Cr–(3.5–5.5)Mo–6W alloys than in the 10Cr–2.5Mo–6W alloy. Cr_2_O_3_ is a p-type semiconductor [37] with a high concentration of metal vacancies. When the higher valence metal is incorporated in the Cr_2_O_3_, the metal vacancies increase, and the metal diffusion rate through the Cr_2_O_3_ layer is enhanced. However, if Mo in the scale forms separate oxides such as MoO_2_ or MoO_3_ because of an even higher Mo content, the vacancy concentration will not increase further. It should be noted that MoO_3_ is also volatile [13,14,15,16,17]. The increase in metal vacancy and the increase in volatilization, as mentioned above, could be the reason for the higher porosity of the Cr oxide layer in alloys with a higher Mo content. However, it was observed that more Al_2_O_3_ lumps were formed and some of them were connected, although not completely continuous, in the alloy with a higher Mo content. It is considered that the higher oxygen diffusion through the porous Cr oxide layer formed in high Mo alloys promotes the internal oxidation of Al. Al_2_O_3_ provides protection against further oxidation. Consequentially, Mo decreased the mass gain at 1000 °C as shown in Figure 3b, when the Mo content increased up to 3.5 wt.%.

Figure 6a, f and g show the effects of W on the oxide structure. Al_2_O_3_ formed more in the 10Cr–3.5Mo–6W alloy and the thickness and porosity of Cr_2_O_3_ were also increased as the W content increased. These increments are shown in the figures for the alloys with 4–6 wt.%. W is thought to exert similar but more complicated effects than Mo, as it is a high valence element which forms a volatile oxide [38] and has a low diffusion rate because of its large size. W causes low oxygen activity due to its high valence and can result in the suppression of CrO_3_ formation. This proposition is also supported by Huang et al. [19], who reported that W suppresses the volatilization of Cr oxide in from Ni–Cr–W alloys at temperatures higher than 1100 °C. However, WO_3_ itself has volatility, making pores in the scale. The large size of W retards the diffusion of other elements and the formation of Al_2_O_3_ can also be retarded. The effects of W on various volatilizations and diffusion rates of elements are not explicitly studied in this study. We can only presume that these counteractions summed up and caused almost zero dependence of mass gain on the W content, as Figure 3b shows.

Park et al. [29] previously reported that the main effects of the alloying elements were as that Cr and Mo increased mass gain slightly, but W did not affect the mass gain by oxidation of Ni-based superalloy at 1000 °C. According to their reports for the interactions between elements, the effects of Cr and Mo were rarely affected by other elements. One exception is that the increasing effect of Cr and Mo were more prominent with alloys with 3 wt.% Al content than with alloys with higher Al content. In contrast, W slightly reduced the mass gain for the 3 wt.% alloys and slightly raised the mass gain of alloys with Mo content over 2.5 wt.%. Considering that the content of Al was 3 wt.% and that of Mo was 3.5 wt.% in the comparative study of this work, the effects of W appear to be compensated by the interactions of the Al and Mo contents.

## 4. Conclusions

The effects of Cr, Mo, and W on the high temperature oxidation of Ni-based superalloys were examined by cyclic oxidation tests at 850 ° and 1000 °C.

The oxidation scale consists mainly of Cr_2_O_3_ and NiCrO_4_ with some NiO, TiO_2_, and CrTaO_4_. Al_2_O_3_ was formed under the scale discontinuously.

The mass gain after 20 cycles of oxidation tests was increased by increasing Cr, Mo, and W at 850 °C. The alloys with higher Cr, Mo, or W contents showed oxide structures with less Al_2_O_3_. Al_2_O_3_ appears to have an important role in protecting the alloy from oxidation even at this temperature as the mass gain was lower for the alloys with more Al_2_O_3_, although the dense Cr_2_O_3_ layer is present.

The oxidation rates were much higher at 1000 °C than at 850 °C. The mass gain was increased by adding Cr but reduced by adding Mo. W did not show a clear effect. These phenomena are explained by the complex effects of the volatilization of Cr, Mo, and W oxides at this temperature, the high valence of Mo and W reducing metal vacancy in the Cr_2_O_3_ layer, and the retarded diffusion due to the large size of W.

## Figures and Tables

**Table 1:** Nominal composition of alloys used in this study.

Sample no.	Co	Cr	Mo	W	Al	Ti	Ta	C	B	Ni
8Cr–3.5Mo–6W	9.5	8	3.5	6	3	5	3	0.1	0.01	Bal.
10Cr–2.5Mo–6W	9.5	10	2.5	6	3	5	3	0.1	0.01	Bal.
10Cr–3.5Mo–4W	9.5	10	3.5	4	3	5	3	0.1	0.01	Bal.
10Cr–3.5Mo–6W	9.5	10	3.5	6	3	5	3	0.1	0.01	Bal.
10Cr–3.5Mo–8W	9.5	10	3.5	8	3	5	3	0.1	0.01	Bal.
10Cr–4.5Mo–6W	9.5	10	4.5	6	3	5	3	0.1	0.01	Bal.
10Cr–5.5Mo–6W	9.5	10	5.5	6	3	5	3	0.1	0.01	Bal.
12Cr–3.5Mo–6W	9.5	12	3.5	6	3	5	3	0.1	0.01	Bal.
